# Standardizing CRISPR-Cas13 knockdown technique to investigate the role of cdh2 gene in pituitary development through growth hormone expression and transcription factors

**DOI:** 10.3389/fendo.2024.1466638

**Published:** 2024-10-10

**Authors:** Bianca Helena Ventura Fernandes, Mara S. Junqueira, Calum MacRae, Luciani R. Silveira de Carvalho

**Affiliations:** ^1^ Laboratory of Hormones and Molecular Genetics (LIM/42), Developmental Endocrinology Unit, Discipline of Endocrinology and Metabolism, Hospital das Clinicas da Faculdade de Medicina da Universidade de São Paulo, São Paulo, Brazil; ^2^ Zebrafish Facility, Technical Support Directorate for Teaching and Research, Faculty of Medicine, University of São Paulo, São Paulo, Brazil; ^3^ Department of Medicine, Harvard Medical School, Boston, MA, United States; ^4^ Center for Translational Research in Oncology, Cancer Institute of the State of São Paulo, Faculty of Medicine, University of São Paulo, São Paulo, Brazil

**Keywords:** zebrafish, genome editing, CRISPR/Cas, knockdown, hypopituitarism

## Abstract

**Introduction:**

Congenital hypopituitarism (CH) is characterized by the deficiency of pituitary hormones. Among CH patients, 85% lack a molecular diagnosis. Whole Exome Sequencing (WES) identified a homozygous variant (c.865G>A, p.Val289Ile) in the *CDH2* gene, responsible for N-Cadherin production, crucial for cell-cell adhesion. Predicted to be likely pathogenic, the variant was found in a patient deficient in *GH*, *TSH*, *ACTH*, and *LH/FSH*. Its impact on cell adhesion was confirmed in L1 fibroblast cell lines.

**Objective:**

Create a *cdh2* knockdown in zebrafish for investigating its role in pituitary development through growth hormone and transcription factors expression.

**Methods:**

Utilized pET28B-RfxCas13d-His plasmid for Cas13 mRNA production via *in vitro* transcription, guiding Cas13 to *cdh2* with three RNAs. Injected the complex into single-cell embryos for analysis up to 96 hpf. Assessed gene expression of *cdh2*, *prop1*, *pit1*, and *gh1* using RT-qPCR. Evaluated cdh2 protein expression through the western blot technique.

**Results:**

Knockdown animals displayed developmental delay. The *cdh2* expression decreased by 75% within 24 hours, rebounded by 48 hours, and reached wild-type levels by 96 hpf. *gh1* expression decreased at 48h but increased by 96 hpf, aligning with WT. No significant differences in *prop1* and *pit1* expression were observed.

**Conclusion:**

Our findings underscore *cdh2*’s role in pituitary development and hormonal regulation, offering insights for developmental biology research.

## 1 Introduction

Pituitary organogenesis is orchestrated by the intricate interplay of signaling pathways, leading to the synchronized expression of transcription factors that are spatially and temporally regulated, maintaining a delicate balance between cell proliferation and differentiation (1). Disruptions in these genetic networks can lead to congenital hypopituitarism, which is characterized by a deficiency of one or more pituitary hormones (2).

The incidence of Congenital hypopituitarism (CH) is approximately 1 in 16.000 births (3, 4). A meta-analysis of 21 studies identified a mutation frequency of 12.4% in critical genes including *PROP1^^1^^*, *POU1F1, HESX1, LHX3*, and *LHX4* (5). The mutation detection rate in sporadic cases (11.2%) was notably lower compared to familial cases (63%). The CH exhibits significant genetic heterogeneity, with mutations in approximately 30 genes implicated in the disorder as of 2016 (6). Notable genetic discoveries include ARNT2, IGSF1, and KCNQ1 in familial cases or offspring from consanguineous unions identified via whole exome sequencing (WES) (7–9). Advances in WES and detection of copy number variations have further expanded the implicated gene pool, adding 37 more genes with varying degrees of pathogenicity.

Recently, WES of a patient from a Brazilian consanguineous family with CH revealed a novel homozygous variant in the *CDH2* (N-cadherin) gene, proposing it as a potential candidate gene. Functional assays demonstrated that this variant diminishes cell aggregation, thereby implicating *CDH2*’s role in pituitary development and meriting further translational research (10).

Zebrafish is an advantageous model for gene function studies due to its significant genetic homology with humans (approximately 70%), manageable size, and cost-effective maintenance. Their external fertilization simplifies genetic manipulation (11). CRISPR-Cas9 genome editing has become essential for elucidating gene functions, allowing targeted mutations in specific genes, resulting in frame-shift mutations and loss of gene function (12–14). However, *cdh2* knockout in zebrafish embryos causes severe developmental abnormalities and early lethality, preventing characterization of complete gene absence.

To model the patient’s variant without completely abolishing gene function, we used a single-base editing approach employing a plasmid that includes a cytidine deaminase fused to Cas9 nickase. This tool enables the direct conversion of C to T or G to A in the DNA sequence (15). Despite injecting 63 animals for single-base editing of the *cdh2* gene, only one animal (1.58%) exhibited the desired amino acid alteration, but different location compared to the Human genetic alteration, indicating the low efficiency of the technique and the complexity in establishing variant-specific lineages (data not showed).

The knockdown model, utilized through RNA interference (RNAi) techniques, is a post-transcriptional gene regulation process. Small RNA effector molecules, such as microRNAs (miRNAs) or externally introduced small interfering RNAs (siRNAs), selectively regulate mRNA expression by either inducing degradation or inhibiting translation (16, 17). This model is particularly advantageous when the gene of interest is crucial to developmental processes, where a complete absence of the protein it encodes would result in lethality, as is often the case with knockout techniques. The knockdown method enables a controlled reduction in gene expression, preserving some gene function, which is critical for studies where complete loss-of-function cannot be tolerated or is not the focus (16). It provides significant insights into the gene’s role and its biological interactions, enhancing our understanding of the molecular mechanics driving physiological and pathological states.

Despite the efficacy of morpholino and antisense oligonucleotides in gene silencing, issues such as cost, toxicity, and off-target effects have restricted their widespread use in systematic screenings (18). Morpholinos (MOs) can block translation or RNA splicing but often lead to nonspecific effects separate from target RNA perturbation (19, 20). Moreover, phenotypes induced by MOs can differ significantly from those obtained by genetic mutation, complicating the interpretation of results (21). These discrepancies are sometimes attributed to genetic compensation (22), emphasizing the need for rigorous controls (23).

Conversely, the CRISPR-RfxCas13d (CasRx) system has proven to be a highly efficient, precise, and cost-effective method for gene knockdown (16, 24). RfxCas13d does not have nucleotide sequence restrictions or protospacer flanking site (PFS) requirements in eukaryotic cells (16, 25), allowing for the targeting of any sequence, including untranslated regions, alternative splice sites, and long noncoding RNAs. Targeting mRNA levels with Cas13d results in immediate degradation, facilitating validation through western blot analysis, quantitative RT-qPCR, or RNA sequencing.

Given the excessively detrimental effects observed with cdh2 gene knockout, which led to severe malformations and impeded characterization (10), and the low efficiency of the knockin approach, which failed to yield a substantial number of animals with the desired variant for characterization, we employed the CRISPR/Cas13 knockdown technique. This method aims to reduce the expression of the protein encoded by the cdh2 gene by approximately 75%. The use of synthetic gRNAs and Cas13d mRNA can produce more pronounced maternal phenotypes, likely due to their earlier activity following injection (16).

The goal of this study was to establish a cdh2 gene knockdown model in zebrafish using the CRISPR-RfxCas13d system to clarify the relationship between cdh2 and the expression of gh1 (Growth Hormone 1) and its transcription factors to determine if cdh2 influences pituitary development and the expression of this hormone.

## 2 Materials and methods

### 2.1 Zebrafish maintenance and husbandry

Experiments involving animals were conducted at the University of São Paulo Medical School. The study protocols were sanctioned by the University’s Ethics Committee on Animal Use (CEUA), approval number 1675/2021. The zebrafish were housed in 3.5-liter aquariums provided by Altamar/Brasil, with each containing 15 adult fish. Environmental conditions were rigorously controlled: pH ~7.5, conductivity between 500-700 μS/cm, and water temperature maintained at 28°C ± 1. A photoperiod regimen of 14 hours light and 10 hours darkness was established. The zebrafish were sustained at the Central Animal Facility of the Medical School, USP, and received Gemma micro commercial feed (Skretting, a Nutreco company^®^) three times daily, the feed size commensurate with the age of the fish, ranging from 75 to 300 microns.

### 2.2 sgRNA design

To pinpoint the most accessible regions for sgRNA targeting, we performed in silico analysis using the RNAfold software (Lorenz et al., 2011). Inputting the cDNA sequence into the software, we opted for settings that would minimize Free Energy (MFE) and identify Partition Function while avoiding isolated base pairs. We visualized RNA secondary structure plots to locate 22–23 nucleotide protospacers with high accessibility, indicated by a low probability of base-pairing, within the Coding Sequence (CDS). We selected three distinct protospacers, each spaced at least 50 nucleotides apart throughout the transcript to avert overlap of guide RNAs (gRNAs) (**Table 1**). This strategy was integral for the successful identification of prime target sites within the mRNA for precise and effective silencing. A guide was manufactured as a positive control for the tbxta gene as described by Kushawah G at al, 2020 (26). The protospacers were synthesized as single-stranded oligonucleotides by Thermo Fisher.

**Table 1:** Sequences of sgRNAs for the *cdh2* gene (1, 2, and 3), Positive Control (tbxta), and Universal Primer.

sgRNA	Sequences
1. *cdh2*	**CGGAGGCGTGATGCTGGGGCTTC**GTTTCAAACCCCGACCAGTT
2. *cdh2*	**TCCAGTCTGATAAGGACAAGAGC**GTTTCAAACCCCGACCAGTT
3. *cdh2*	**TAATATCACAGCAGTAGACGGAG**GTTTCAAACCCCGACCAGTT
*Tbxta* (Positive Control)	**CTTTGCTGAAAGATACGGGTGCT**GTTTCAAACCCCGACCAGTT
Universal Primer to IVT	TAATACGACTCACTATAGGAACCCCTACCAACTGGTCGGGGTTTGAAAC

In bold are the target DNA sequences specific to the gene.

### 2.3 Plasmid transformation

The pT3TS-RfxCas13d-HA plasmid, obtained from Addgene (Plasmid #141320, http://www.addgene.org/141320/), was propagated according to Addgene’s specifications. We cultured ampicillin-resistant bacteria on brain heart infusion (BHI) agar medium supplemented with 100 μg/ml ampicillin. Post a 24-hour incubation at 37°C, a single colony was propagated in liquid BHI medium for another 24 hours at 37°C. Following centrifugation (10000g for 30 seconds), we discarded the supernatant and resuspended the pellet in a solution1 containing 25 mM Tris-HCl, 50 mM glucose, and 10 mM EDTA, followed by the addition of solution 2 (0.2N NaOH and 1% SDS). After 5 minutes on ice incubation, we added a cold solution 3 (120 mL 5M Potassium Acetate, 23 mL acetic acid, and 57 mL MilliQ water), followed by another 5 minutes ice incubation. Post-centrifugation (12000g for 5 minutes), the supernatant was mixed with cold 100% ethanol and 3M sodium acetate and incubated at -20°C and centrifuged at 4°C for 30 minutes the next day. The resultant pellet was resuspended in TE buffer (10 mM Tris-HCl, 1 mM EDTA).

### 2.4 *In vitro* transcription of plasmid and RNA guides

Two micrograms of the plasmid were digested with BamHI restriction enzyme for 4 hours, and successful digestion was confirmed via agarose gel electrophoresis by using 1μL of the reaction applied to a 1.5% agarose gel. The digested sample was purified using a commercial kit (TIANquick mini purification Kit) and then quantified using nanodrop spectrophotometer. For *in vitro* transcription, we employed the T7 mMessage mMachine kit. The transcribed RNA was verified on an agarose gel, purified using the gene art precision kit (thermofisher), and quantified on nanodrop spectrophotometer. The primers for the sgRNAs were synthesized in the form of DNA templates, and *in vitro* transcription was performed using a commercial kit, as described in the protocol by Kushawah et al., 2020 (16).

### 2.5 gRNA microinjection

After producing the sgRNAs and transcribing the plasmids, the samples were quantified using a Nanodrop spectrophotometer, and the injection mix was prepared with 300 ng/μL of plasmid RNA and 900 ng/μL of total sgRNA (300 ng of each sgRNA). The injection needles were prepared from borosilicate glass capillaries (Sutter Instrument, USA) using a micropipette puller (Sutter Instrument, USA). The needles were filled with 5 μL of the knockout, knockin, or knockdown mix using a microloader (Eppendorf). Next, the needle was placed in a holder connected to a pneumatic microinjection pump (Harvard Apparatus, USA).

Injections were performed at 6.3X magnification under a stereomicroscope (SMZ 745, Nikon, Germany). The injection volume was calibrated using a micrometer ruler (Bresser 1/10 mm), according to the formula for the volume of a sphere (V = 1/6πd^3), where a sphere diameter of 1 bar corresponds to an injection volume of 1 nL.

To initiate the injection, the embryos were placed in a Petri dish with an agarose mold (WPI, USA) to prevent them from slipping under the needle pressure. 1 nL of the respective mix was injected into the single-cell stage embryo.

### 2.6 Gene expression analysis by RT-qPCR

Gene expression levels of *cdh2*, *gh1*, *prop1*, and *pit1* were assessed by Real-Time PCR in zebrafish embryos at key developmental stages (24, 48, 72, and 96 hpf) we utilized 20 embryos per pool and performed 5 distinct pools. We utilized the endogenous gene ef1a as a normalization control. RNA was extracted following the Trizol method and stored at -80°C after elution in Milli-Q water. Total RNA was quantified with a NanoPhotometer P-300 (Implen, Munich, Germany), and cDNA was synthesized from 1000 ng of total RNA using the High-Capacity cDNA Reverse Transcription kit (Applied Biosystems, Foster City, CA, USA), according to the manufacturer’s instructions. Primers used are detailed in **Table 2**. cDNA samples underwent PCR using SYBR™ Green PCR Master Mix (Applied Biosystems, Foster City, CA, USA) and amplified in an AriaMx Real-Time PCR System (Agilent, Santa Clara, CA, USA). Gene expression was normalized against wild-type controls and the least variable endogenous gene. The relative quantification was calculated using the 2^-ΔΔCT method by Livak and Schmittgen (2001) (27) where ΔCT is the difference in expression between the target and endogenous control genes, and ΔΔCT is the ΔCT difference between the mutant and wild-type samples for the same developmental period. Each sample was processed in triplicate. The nucleotide sequences of the primer pairs used are described in **Table 2**. The primers for the ef1a, pit1 (28), and gh1 (29) genes have been previously reported in the literature. The primers for amplifying the prop1 and cdh2 genes were designed in our laboratory.

**Table 2:** Gene expression primers.

Oligo	Forward 5’ to 3’	Reverse 5’ to 3’
*ef1a*	CGTCTGCCACTTCAGGATG	TGTCTCCAGCCACATTACCA
*cdh2*	ATGAATGACAACCGGCCAGA	ACATGTTGGATGAGGGGCTC
*prop1*	GCACGAGCAAGACCATACCCT	CTGCTTGGCACGACGGTTCT
*pit1*	AAACCAATGTGGGTGAAGCTCTGG	AGCCATTTCGCCAGGATGGATTTG
*gh1*	CCTCTGTCGTTCTGCAACTC	ACTCCCAGGATTCAATGAGG

### 2.7 Western blot analysis

The presence of cdh2 protein in wild-type and knockdown samples was determined by Western blot. We collected pools of 30 embryos at 24 hpf and 96 hpf, and protein extraction was carried out. Briefly, samples were lysed in 100 μL of RIPA buffer (ThermoFisher Scientific, USA) and protease inhibitor (Sigma, St. Louis, USA). Each sample was quantified using the Pierce BCA Protein Assay kit (Thermo Fisher Scientific, Massachusetts, USA). Equal amounts of protein (50 μg) dissolved in SDS sample buffer were loaded onto 12% SDS-PAGE (sodium dodecyl sulfate-polyacrylamide gel electrophoresis) gel wells and subjected to electrophoresis in a mini-gel tank (Bio-Rad, Brazil) for 2 hours at 120V. Following electrophoresis, proteins separated in the gel were transferred to a nitrocellulose membrane. The membranes were then blocked with 5% skim milk (Molico, Brazil) diluted in TBST (1M Tris-HCL, 3M NaCl, and 0.2% Tween 20) for 2 hours. After blocking, the membrane was incubated in a solution of 5% skim milk and PBST with primary antibody Anti CDH2 (ABCAM) diluted at 1:500 and/or Anti-Actin (SIGMA) diluted at 1:5000 and incubated at 4°C overnight with agitation. Three quick washes with PBS were performed after incubation, followed by three 10-minute washes with PBST. Then, the membrane was incubated with secondary anti-Rabbit antibody conjugated with HRP diluted at a concentration of 1:5000 in PBST for 2 hours at 4°C. The antigen-antibody immunocomplex was visualized with SuperSignal West Pico Plus chemiluminescent substrate (Thermo Scientific, Rockford, USA). Band intensities were visualized using an imaging capture system (UVITEC, Cambridge, UK).

### 2.8 Phenotypic analysis

The animals were examined under a stereomicroscope (Nikon SMZ800) at 48 hours post-fertilization, and their morphology was analyzed.

### 2.9 Statistical analysis

To compare two groups, both parametric and non-parametric tests were applied based on data distribution. For normally distributed data, an unpaired Student’s t-test was used. For non-normally distributed data, a Mann-Whitney U test was applied. Statistical significance was set at p < 0.05. All statistical analyses were performed using GraphPad Prism software, with results presented as mean ± standard error of the mean (SEM).

## 3 Results

### 3.1 Technique efficiency

**Figure 1:**
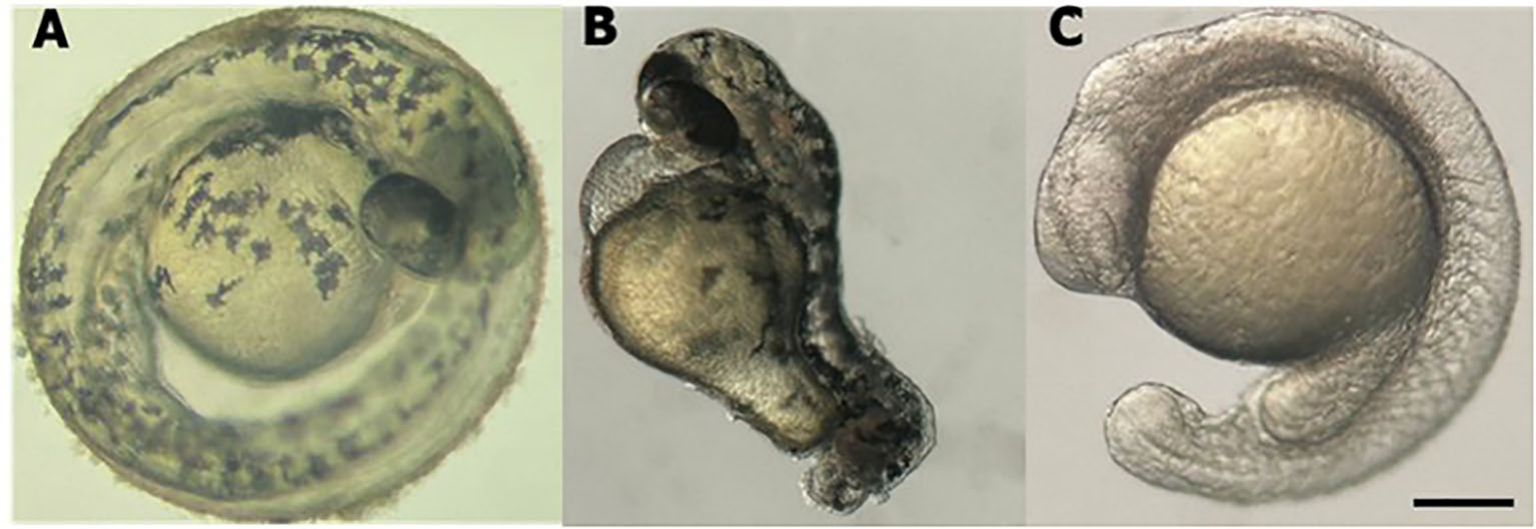
Stereomicroscope image of larval zebrafish at 48 hpf. **(A)** WT l. **(B)** *tbxta* gene knockdown. **(C)** *cdh2* gene knockdown. Scale bar = 200 μm.

The efficacy of the technique was evaluated by observing the phenotypes of the animals and measuring the expression levels of the *cdh2* gene in *cdh2* knockdown animals. For greater reliability of the technique and better visualization of its efficiency, we performed a positive control with guides for the tbxta gene (no tail), where the animal is formed without the tail, these guides are provided in the pipeline by Kushawah G, et al., 2020 (16). Stereomicroscopic analysis revealed that animals injected with sgRNA for the *tbxta* gene (a positive control) exhibited tail shortening, which confirmed the effectiveness of the method. Conversely, animals injected with sgRNA targeting the *cdh2* gene mRNA displayed developmental deficiencies like head malformation, and developmental delay. These phenotypes, although milder, were similar to those observed in *cdh2* knockout animals (10) (**Figure 1**).

**Figure 2:**
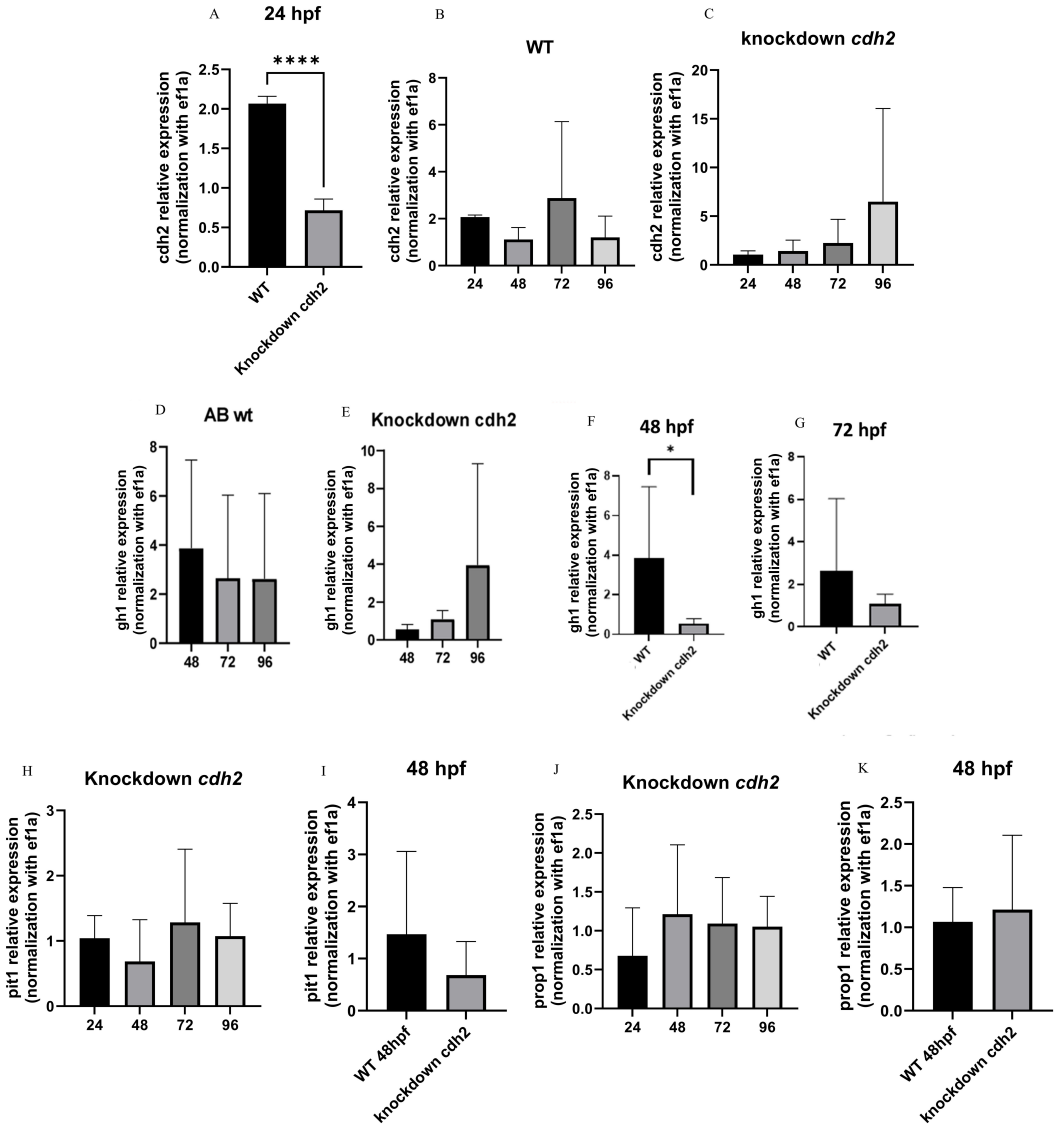
*G*ene expression graph **(A)** *cdh2* at 24 hpf in WT and *cdh2* knockdown larvae; (***p.<0,0001) **(B)** *cdh2* temporal expression in WT larvae from 24 to 96 hpf; **(C)** *cdh2* temporal expression in *cdh2* knockdown larvae from 24 to 96 hpf; **(D)** Temporal expression of *gh1* at 48, 72, and 96 hpf in WT larvae. **(E)** Temporal expression of *gh1* at 48, 72, and 96 hpf in *cdh2* knockdown larvae. **(F)** Expression of *gh1* in *cdh2* knockdown larvae compared to WT at 48 hpf (*p.<0.05). **(G)** Expression of *gh1* in *cdh2* knockdown larvae compared to WT 72 hpf (NS). **(H)** *pit1* temporal expression in *cdh2* knockdown larvae from 24 to 96 hpf. **(I)** *pit1* expression at 48 hpf in *cdh2* knockdown larvae compared to WT larvae (NS). **(J)** *prop1* temporal expression in *cdh2* knockdown larvae from 24 to 96 hpf. **(K)** *prop1* expression at 48 hpf in *cdh2* knockdown larvae compared to WT (NS).

In addition to the phenotypic analysis, the expression of *cdh2* in knockdown animals was investigated to validate the efficacy of the technique in reducing gene expression. A 75% decrease in *cdh2* gene expression was observed at 24 hpf (**Figure 2A**) in comparison to wild-type (WT) animals. From 48 to 96 hpf, there was a progressive restoration of *cdh2* expression in knockdown animals (**Figure 2C**), as anticipated for the knockdown approach, whereas in WT animals, gene expression remained stable until 96 hpf (**Figure 2B**).

### 3.2 Analysis of cdh2 protein expression in knockdown animals

**Figure 3:**
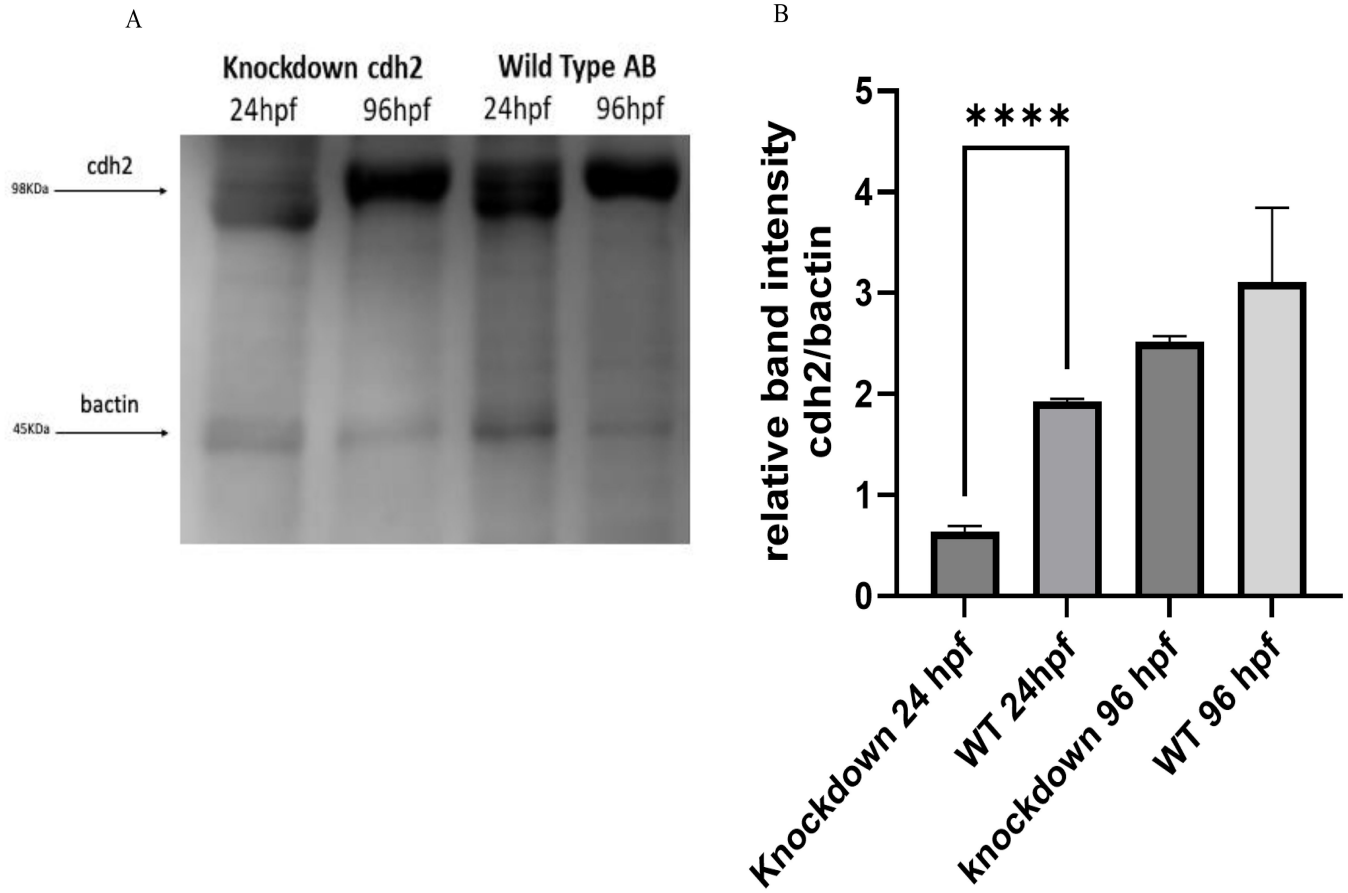
**(A)** Protein expression of cdh2 (n-cadherin) assessed by Western blot technique in WT animals at 24 and 96 hpf, and in animals subjected to knockdown of the *cdh2* gene at 24 and 96 hpf. **(B)** Relative band intensities of Western Blots loaded with 50 µg of pooled protein from larvae. The immunoblot band density in each lane was normalized against the density of the endogenous protein band (β-actin) (****p.<0,0001).

Western blot analysis supported the gene expression results obtained by RT-qPCR, confirming the reduction of the cdh2 protein in knockdown animals. At 24 hpf, the protein expression was reduced by 64% compared to WT animals, as shown in **Figures 3A, B**. Notably, at 96 hpf, protein expression in knockdown animals appeared to be more substantial than in WT animals, suggesting a compensatory overexpression following the initial suppression. These results corroborate the effectiveness of the knockdown approach in significantly reducing the expression of the cdh2 protein initially, followed by a recovery of protein production over time. This recovery suggests that the knockdown not only transiently blocked *cdh2* gene expression but also permitted a full restoration of protein production, elucidating the role of the *cdh2* gene and the n-cadherin protein in development and validating the reversibility of the knockdown method.

### 3.3 Gene expression of hormones and transcription factors

We examined the effect of *cdh2* suppression on embryonic hormone production by analyzing the gene expression of markers such as gh1 (growth hormone), prop1 (PROP Paired-Like Homeobox 1), and pit1 (POU class 1 homeobox 1) through RT-qPCR. The temporal expression pattern of *gh1* was inversely related to WT animals, where expression in WT began higher and decreased over time, whereas in knockdown animals, it started lower and increased (**Figures 2D, E**). The production of *gh1* was statistically lower at 48 hpf in knockdown animals compared to WT (**Figure 2F**). At 72 hpf, the trend was toward a decrease compared to WT (**Figure 2G**), and at 96 hpf, when *cdh2* production paralleled WT, *gh1* expression recovered to levels comparable to WT (**Figures 2D, E**).

Regarding the markers *prop1* and *pit1*, analyses were initiated at 24 hpf and extended up to 96 hpf, with evaluations conducted every 24 hours. In **Figure 2H**, we can observe the temporal expression of *pit1* in the *cdh2* knockdown group, showing a trend toward decreased expression at 48 hpf, although it was not statistically different from WT animals (**Figure 2I**). The expression of *prop1* remained constant in *cdh2* knockdown animals from 24 to 96 hpf (**Figure 2J**) and was equivalent to WT at 48 hpf (**Figure 2K**) when the *gh1* was decreased.

## 4 Discussion

The CRISPR-RfxCas13d system offers a significant advantage over traditional gene modulation techniques, particularly when studying essential genes in embryonic animal models (30). Unlike knockout methods, which can lead to lethality in crucial developmental genes, CRISPR-RfxCas13d allows for targeted mRNA degradation without completely abolishing gene function, thereby avoiding the complications of early lethality (31). Additionally, compared to knock-in techniques, which are notoriously inefficient, expensive, and time-consuming—often requiring up to two years to establish a desired zebrafish lineage with a success rate of only about 3%—the CRISPR-RfxCas13d system is far more efficient, cost-effective, and quicker to implement (16, 32). This approach also avoids the burden on endogenous cellular machinery typically seen with RNAi, preserving normal cellular functions, particularly in models like teleost fish where RNAi is less effective (33).

Moreover, the CRISPR-RfxCas13d system provides a versatile platform for targeting a wide range of RNA sequences without nucleotide sequence restrictions, making it highly adaptable to various research needs. It demonstrates minimal off-target effects and does not induce significant physiological responses, apart from the intended developmental phenotypes. This system’s ease of use, combined with its flexibility and low cost, makes it an excellent choice for large-scale screening studies, particularly for maternal factors involved in early development. Furthermore, the ability to utilize this system in transgenic lines opens up new possibilities for studying gene function in a tissue-specific manner or in cases where gene disruption would otherwise be lethal. These advantages not only make CRISPR-RfxCas13d an effective tool for research but also pave the way for potential therapeutic applications, such as targeting viral RNAs, as demonstrated in recent studies (16).

The present study aimed to knockdown the *cdh2* gene due to the obstacles commonly encountered with knockout methodologies, particularly their potential lethality when targeting genes that are critical for development. Additionally, knock-in techniques are known to be inefficient and require significant effort and financial resources to achieve reproducible results. With current technologies, obtaining a zebrafish with the desired variant through knockin techniques has an efficiency of only about 3%, and it can take long time to establish the desired lineage (32, 34).

By applying the knockdown strategy in our study, we achieved a targeted reduction in *cdh2* gene expression by approximately 75%. This approach allows for the partial reduction of gene function, thereby avoiding the lethality associated with complete knockouts while still providing valuable insights into gene function. To further validate the effectiveness of our knockdown approach, we conducted a positive control experiment in accordance with the protocol established by Kushawah G, et al., 2020 (16).

The efficiency of the knockdown sgRNA was evaluated through real-time PCR gene expression analysis, complemented by Western blot analyses to assess protein expression. These combined approaches confirmed the successful implementation of the knockdown technique, demonstrating its advantages over more traditional gene-editing methods, particularly when considering the challenges and limitations associated with knock-in and knockout approaches.

Phenotypic analysis of animals with reduced *cdh2* expression exhibited abnormal morphological features, such as developmental delay, and blurred distinction between the mesencephalon and rhombencephalon. These observations agree with those of Angela and Mark M. Voigt, who reported similar phenotypes in *cdh2 morpholino (MO)* knockdown zebrafish (35). This concordance underscores the role of *cdh2* in neural circuit formation and underpins its significance in proper neural development.

Gene expression analysis of hormone-related genes revealed a reduction in *gh1* expression concurrent with *cdh2* suppression. This finding aligns with *in vitro* evidence showing that n-cadherin suppression in fetal pituitary cultures precipitates a swift decline in GH secretion, emphasizing *cdh2*’s specific regulatory role in GH synthesis (36).

Furthermore, the unaltered expression of *pit1* and *prop1* in *cdh2* knockdown specimens suggests a specific association between *cdh2* and *gh1* secretion. This implies that the intra pituitary axis and intercellular communication among hormone-secreting pituitary cells may serve as additional regulatory mechanisms for hormone secretion. These insights shed light on *cdh2*’s distinct function in the modulation of hormonal secretion and its significance within pituitary gland development and endocrine physiology.

These discoveries greatly enrich our comprehension of developmental mechanisms, setting the stage for further exploration of *cdh2*’s impact on various facets of embryonic development and organ morphogenesis. By elucidating the regulatory effects of *cdh2* on hormonal balance and its link to pituitary organogenesis, we furnish new perspectives on the intricate genetic and molecular interplay fundamental to vertebrate development.

The findings presented herein affirm that the CRISPR/Cas13 knockdown technique is a viable and promising method for functional genomic studies pertinent to developmental biology. It enables selective suppression of gene expression, offering a refined approach to dissect the roles of specific genes in essential biological processes, circumventing the difficulties inherent in creating complete knockout models. This advancement significantly augments the developmental biologist’s research repertoire, fostering novel avenues for investigating regulatory pathways integral to embryonic development and organogenesis.

## Data Availability

The original contributions presented in the study are included in the article/supplementary material. Further inquiries can be directed to the corresponding author.

## Footnotes

1 Explanatory footnote for publication: The symbols for genes and proteins were formatted according to the genetic and protein nomenclature guidelines established by the "Gene Nomenclature and Requirements" of Oxford Academic. The spelling adheres to specific standards for each species: human genes are denoted in uppercase and italicized, mouse genes in italic with only the first letter capitalized, and zebrafish genes in italic with all lowercase letters. Protein symbols follow the same capitalization rule but are not formatted in italics.

## References

[1] Sönksen PH Salomon F Cuneo R. Metabolic effects of hypopituitarism and acromegaly1. Horm Res. (1991) 36:27–31. doi: 10.1159/000182184 1806480

[2] Miljić D Popovic V. Metabolic syndrome in hypopituitarism. In. (2018) 49: 1–19. doi: 10.1159/000485997 29895033

[3] Mullis PE. Genetics of ısolated growth hormone deficiency. J Clin Res Pediatr Endocrinol. (2010) 2:52–62. doi: 10.4274/jcrpe 21274339 PMC3014602

[4] Castets S Thomas-Teinturier C Villanueva C Amsellem J Barat P Brun G. Diagnosis and management of congenital hypopituitarism in children. Arch Pédiatrie. (2024) 31:165–71. doi: 10.1016/j.arcped.2024.01.003 38538470

[5] Jakobsen LK Jensen RB Birkebæk NH Hansen D Christensen AMR Bjerrum MC. Diagnosis and incidence of congenital combined pituitary hormone deficiency in Denmark—A national observational study. J Clin Endocrinol Metab. (2023) 108:2475–85. doi: 10.1210/clinem/dgad198 PMC10505542 37043518

[6] Davis SW Castinetti F Carvalho LR Ellsworth BS Potok MA Lyons RH. Molecular mechanisms of pituitary organogenesis: In search of novel regulatory genes. Mol Cell Endocrinol. (2010) 323:4–19. doi: 10.1016/j.mce.2009.12.012 20025935 PMC2909473

[7] Nishigaki S Hamazaki T Fujita K Morikawa S Tajima T Shintaku H. A Japanese family with central hypothyroidism caused by a novel IGSF1 mutation. Thyroid. (2016) 26:1701–5. doi: 10.1089/thy.2016.0005 27762734

[8] Tommiska J Känsäkoski J Skibsbye L Vaaralahti K Liu X Lodge EJ. Two missense mutations in KCNQ1 cause pituitary hormone deficiency and maternally inherited gingival fibromatosis. Nat Commun. (2017) 8:1289. doi: 10.1038/s41467-017-01429-z 29097701 PMC5668380

[9] Webb EA AlMutair A Kelberman D Bacchelli C Chanudet E Lescai F. ARNT2 mutation causes hypopituitarism, post-natal microcephaly, visual and renal anomalies. Brain. (2013) 136:3096–105. doi: 10.1093/brain/awt218 PMC3784281 24022475

[10] Ferreira NGBP Madeira JLO Gergics P Kertsz R Marques JM Trigueiro NSS. Homozygous CDH2 variant may be associated with hypopituitarism without neurological disorders. Endocr Connect. (2023) 12:e220473. doi: 10.1530/EC-22-0473 PMC10388658 37166408

[11] Nayak R Franěk R Šindelka R Pšenička M. Enhancement of zebrafish sperm production via a large body-sized surrogate with germ cell transplantation. Commun Biol. (2023) 6:412. doi: 10.1038/s42003-023-04800-7 37059808 PMC10104805

[12] Kimmel CB. Genetics and early development of zebrafish. Trends Genet. (1989) 5:283–8. doi: 10.1016/0168-9525(89)90103-0 2686119

[13] Saleem S Kannan RR. Zebrafish: an emerging real-time model system to study Alzheimer’s disease and neurospecific drug discovery. Cell Death Discovery. (2018) 4:45. doi: 10.1038/s41420-018-0109-7 PMC6170431 30302279

[14] Westerfield M. The zebrafish book. In: A guide for the laboratory use of zebrafish (Danio rerio), 4th. Univ. of Oregon Press, Eugene (2000).

[15] Qin W Lu X Liu Y Bai H Li S Lin S. Precise A.T to G.C base editing in the zebrafish genome. BMC Biol. (2018) 16:1–8. doi: 10.1186/s12915-018-0609-1 30458760 PMC6247682

[16] Kushawah G Hernandez-Huertas L Abugattas-Nuñez del Prado J Martinez-Morales JR DeVore ML Hassan H. CRISPR-cas13d induces efficient mRNA knockdown in animal embryos. Dev Cell. (2020) 54:805–17. doi: 10.1016/j.devcel.2020.07.013 32768421

[17] Xavier DJ Takahashi P Evangelista AF Foss-Freitas MC Foss MC Donadi EA. Assessment of DNA damage and mRNA/miRNA transcriptional expression profiles in hyperglycemic versus non-hyperglycemic patients with type 2 diabetes mellitus. Mutat Research/Fundamental Mol Mech Mutagenesis. (2015) 776:98–110. doi: 10.1016/j.mrfmmm.2015.01.016 26364207

[18] LaMora A Voigt MM. Cranial sensory ganglia neurons require intrinsic N-cadherin function for guidance of afferent fibers to their final targets. Neuroscience. (2009) 159:1175–84. doi: 10.1016/j.neuroscience.2009.01.049 PMC2667798 19356698

[19] Gentsch GE Spruce T Monteiro RS Owens NDL Martin SR Smith JC. Innate immune response and off-target mis-splicing are common morpholino-induced side effects in xenopus. Dev Cell. (2018) 44:597–610.e10. doi: 10.1016/j.devcel.2018.01.022 29478923 PMC5861998

[20] Joris M Schloesser M Baurain D Hanikenne M Muller M Motte P. Number of inadvertent RNA targets for morpholino knockdown in Danio rerio is largely underestimated: evidence from the study of Ser/Arg-rich splicing factors. Nucleic Acids Res. (2017) 45:9547–57. doi: 10.1093/nar/gkx638 PMC5766196 28934490

[21] Kok FO Shin M Ni CW Gupta A Grosse AS van Impel A. Reverse genetic screening reveals poor correlation between morpholino-induced and mutant phenotypes in zebrafish. Dev Cell. (2015) 32:97–108. doi: 10.1016/j.devcel.2014.11.018 25533206 PMC4487878

[22] El-Brolosy MA Kontarakis Z Rossi A Kuenne C Günther S Fukuda N. Genetic compensation triggered by mutant mRNA degradation. Nature. (2019) 568:193–7. doi: 10.1038/s41586-019-1064-z PMC6707827 30944477

[23] El-Brolosy MA Stainier DYR. Genetic compensation: A phenomenon in search of mechanisms. PLoS Genet. (2017). 13:e1006780. doi: 10.1371/journal.pgen.1006780 28704371 PMC5509088

[24] Wessels HH Méndez-Mancilla A Guo X Legut M Daniloski Z Sanjana NE. Massively parallel Cas13 screens reveal principles for guide RNA design. Nat Biotechnol. (2020) 38:722–7. doi: 10.1038/s41587-020-0456-9 PMC7294996 32518401

[25] Abudayyeh OO Gootenberg JS Essletzbichler P Han S Joung J Belanto JJ. RNA targeting with CRISPR–cas13. Nature. (2017) 550:280–4. doi: 10.1038/nature24049 PMC5706658 28976959

[26] Kushawah G Hernandez-Huertas L Abugattas-Nuñez del Prado J Martinez-Morales JR DeVore ML Hassan H. CRISPR-Cas13d Induces Efficient mRNA Knockdown in Animal Embryos. Dev Cell [Internet]. (2020) 54:805–817.e7. https://linkinghub.elsevier.com/retrieve/pii/S1534580720305876. 32768421 10.1016/j.devcel.2020.07.013

[27] Livak KJ Schmittgen TD. Analysis of relative gene expression data using real-time quantitative PCR and the 2–ΔΔCT method. Methods. (2001) 25:402–8. doi: 10.1006/meth.2001.1262 11846609

[28] Angotzi AR Mungpakdee S Stefansson S Male R Chourrout D. Involvement of Prop1 homeobox gene in the early development of fish pituitary gland. Gen Comp Endocrinol [Internet]. (2011) 171:332–40. https://linkinghub.elsevier.com/retrieve/pii/S0016648011000761. 10.1016/j.ygcen.2011.02.026 21362424

[29] He W Dai X Chen X He J Yin Z. Zebrafish pituitary gene expression before and after sexual maturation. J Endocrinol. (2014) 221:429–40. https://joe.bioscientifica.com/view/journals/joe/221/3/429.xml. 10.1530/JOE-13-0488 24709578

[30] Chen GR Sive H Bartel DP. A seed mismatch enhances argonaute2-catalyzed cleavage and partially rescues severely impaired cleavage found in fish. Mol Cell. (2017) 68:1095–1107.e5. doi: 10.1016/j.molcel.2017.11.032 29272705 PMC5821252

[31] Dong M Fu YF Du TT Jing CB Fu CT Chen Y. Heritable and lineage-specific gene knockdown in zebrafish embryo. Wölfl S. PloS One. (2009) 4:e6125. doi: 10.1371/journal.pone.0006125 19582161 PMC2702085

[32] Prill K Dawson JF. Homology-directed repair in zebrafish: witchcraft and wizardry? Front Mol Biosci. (2020) 7. doi: 10.3389/fmolb.2020.595474 PMC7793982 33425990

[33] Kelly A Hurlstone AF. The use of RNAi technologies for gene knockdown in zebrafish. Brief Funct Genomics. (2011) 10:189–96. doi: 10.1093/bfgp/elr014 21525144

[34] Gaudelli NM Komor AC Rees HA Packer MS Badran AH Bryson DI. Programmable base editing of A•T to G•C in genomic DNA without DNA cleavage. Nature. (2017) 551:464–71. doi: 10.1038/nature24644 PMC5726555 29160308

[35] LaMora A Voigt MM. Cranial sensory ganglia neurons require intrinsic N-cadherin function for guidance of afferent fibers to their final targets. Neuroscience. (2009) 159:1175–84. doi: 10.1016/j.neuroscience.2009.01.049 PMC2667798 19356698

[36] Rubinek T Yu R Hadani M Barkai G Nass D Melmed S. The cell adhesion molecules N-cadherin and neural cell adhesion molecule regulate human growth hormone: A novel mechanism for regulating pituitary hormone secretion. J Clin Endocrinol Metab. (2003) 88:3724–30. doi: 10.1210/jc.2003-030090 12915661

